# Association of HIV infection with clinical and laboratory characteristics of sickle cell disease

**DOI:** 10.1186/s12879-020-05366-z

**Published:** 2020-08-27

**Authors:** André Rolim Belisário, Paula F. Blatyta, Diana Vivanco, Claudia Di Lorenzo Oliveira, Anna Bárbara Carneiro-Proietti, Ester Cerdeira Sabino, Cesar de Almeida-Neto, Paula Loureiro, Cláudia Máximo, Sheila de Oliveira Garcia Mateos, Miriam V. Flor-Park, Daniela de Oliveira Werneck Rodrigues, Rosimere Afonso Mota, Thelma T. Gonçalez, Thomas J. Hoffmann, Shannon Kelly, Brian Custer, for the Recipient Epidemiology and Donor Evaluation Study-III (REDS-III) International Component Brazil, Ester C. Sabino, Cecilia Alencar, Alfredo Mendrone, Cesar de Almeida Neto, Ligia Capuani, Miriam Park, Paula Blatyta, Anna Bárbara de Freitas Carneiro-Proietti, Carolina Miranda Teixeira, Tassila Salomon, Franciane Mendes de Oliveira, Valquíria Reis, Rosemere Afonso Mota, José Wilson Sales, Daniela de Oliveira Werneck, Paula Loureiro, Aderson Araújo, Maria do Carmo Valgueir, Clarisse Lobo, Claudia Maximo, João Eduardo Ferreira, Márcio Katsumi Oikawa, Pedro Losco Takecian, Mina Cintho Ozahata, Rodrigo Muller de Carvalho, Brian Scott Custer, Michael P. Busch, Shannon Kelly, Thelma Therezinha Gonçalez, Donald Brambilla, Liliana R. Preiss, Christopher McClure

**Affiliations:** 1 Fundação Hemominas, Alameda Ezequiel Dias, 321, Belo Horizonte, Minas Gerais 30130-110 Brazil; 2 Disciplina de Ciências Médicas, Faculdade de Medicina da Universidade de São Paulo, São Paulo, SP Brazil; 3 University of California, San Francisco (UCSF), San Francisco, CA USA; 4 Universidade Federal de São João Del Rei, São João del Rei, Minas Gerais Brazil; 5 Faculdade de Medicina (FMUSP) and Instituto de Medicina Tropical, Universidade de São Paulo, São Paulo, Brazil; 6 Fundação Pró-Sangue Hemocentro de São Paulo, São Paulo, SP Brazil; 7 Fundação Hemope, Recife, Pernambuco Brazil; 8 Universidade de Pernambuco, Recife, Pernambuco Brazil; 9 Fundação Hemorio, Rio de Janeiro, Brazil; 10 ITACI, Unidade de Onco-hematologia, Instituto da Criança, HCFMUSP, São Paulo, Brazil; 11 Vitalant Research Institute, San Francisco, USA; 12 UCSF Benioff Children’s Hospital Oakland, Oakland, USA

**Keywords:** Sickle cell disease, HIV, Disease interaction, Risk factor

## Abstract

**Background:**

Sickle cell disease (SCD) is a multisystem disorder characterized by a wide spectrum of clinical manifestations and severity. Studies investigating potential effects of co-morbid human immunodeficiency virus (HIV) and SCD have produced conflicting results, and additional investigations are needed to elucidate whether the interaction between the two disease states might impact both HIV and SCD clinical outcomes. The association of HIV infection with clinical and laboratory characteristics of patients with SCD was assessed.

**Methods:**

This nested case-control study included individuals with SCD with HIV treated at six Brazilian SCD centers. Clinical and laboratory data were abstracted from medical records. HIV positive participants were compared to age, gender, center, and SCD genotype matched HIV negative participants (ratio 1:4). Individual clinical outcomes as well as a composite outcome of any SCD complication and a composite outcome of any HIV-related complication were compared between the two groups.

**Results:**

Fifteen HIV positive participants were included, 12 (80%) alive and 3 (20%) deceased. Most of the HIV positive patients had HbSS (60%; *n* = 9), 53% (*n* = 8) were female, and mean age was 30 ± 13 years. The frequency of individual SCD complications of acute chest syndrome/pneumonia, sepsis/bacteremia, pyelonephritis, ischemic stroke, hemorrhagic stroke, abnormal transcranial Doppler (TCD), and pulmonary hypertension was higher in HIV positive participants when compared to HIV negative, although analyzed individually none were statistically significant. HIV positive participants had significantly higher risk of any SCD complication and of a composite HIV-related complication compared to the HIV negative group (HR = 4.6; 95%CI 1.1–19.6; *P* = 0.04 and HR = 7.7; 95%CI 1.5–40.2; *P* = 0.02, respectively). There was a non-significant trend towards higher risk of any infections in participants with HIV positive (HR = 3.5; 95%CI 0.92–13.4; *P* = 0.07). Laboratory parameters levels were not significantly different in individuals with and without HIV.

**Conclusions:**

In summary, our study in SCD patients shows that those with HIV have an increased risk of any SCD complication and HIV-related complications, as well as a suggestive but not significantly increased risk of infections.

## Background

Sickle cell disease (SCD) is a set of genetic disorders caused by mutations in the *HBB* gene, which encodes the beta-globin chain of hemoglobin [1]. Population estimates propose that every year nearly 300,000–400,000 neonates are born with SCD worldwide, around 75% in sub-Saharan Africa [2]. In Brazil, 3500 newborns are born with SCD annually, though the incidence varies considerably between the states [3]. SCD is a multisystem disorder characterized by a wide spectrum of clinical manifestations and severity. Genetic and environmental factors play a role in the phenotype diversity of SCD, including the occurrence of infections [1].

The World Health Organization data estimates that in 2018 approximately 36.9 million people were infected with human immunodeficiency virus (HIV) worldwide, the majority in the African continent [4]. In Brazil, approximately 860,000 (630,000 – 1,100,000) individuals were living with HIV in 2017 [5]. In the last three decades, advances in diagnosis and treatment have led to considerable progress in the life expectancy of patients with HIV [6], as well as in those with SCD [1]. Both diseases have become chronic illnesses, especially in developed countries. As SCD and HIV are endemic in overlapping geographic areas, including in Brazil, the coexistence of both diseases has been reported [7]. One systematic review suggested that HIV may worsen symptoms of SCD [7]. Both HIV and SCD are independent risk factors for certain outcomes such as stroke, avascular necrosis, pulmonary hypertension, kidney disease and infections. Therefore, it is possible that HIV and SCD may interact to increase the risk of these complications.

Studies investigating potential effects of co-morbid HIV and SCD have produced conflicting results. Some studies found no relationship between HIV infection and clinical manifestations of SCD [8], whereas others found a higher occurrence of infections [9, 10] in individuals with both diseases. A review of the Nationwide Inpatient Databases of the Healthcare Cost and Utilization Project in the US demonstrated that hospitalized children with SCD and HIV had a significantly higher risk of bacterial infection and sepsis than those with SCD without HIV; the length of hospital stay was longer in individuals with SCD and HIV compared to those without HIV (8.0 days vs. 4.3 days, respectively), and the mean charges associated with the hospitalization were also higher ($18,291 vs. $9584) [10]. Another study showed that the incidence of serious invasive pneumococcal infections (meningitis) observed among HIV positive patients with SCD was significantly higher (> 10 times) than that observed among SCD patients without HIV [9]. However, a retrospective study involving 1415 individuals with SCD from Brazil showed no relationship between HIV infection and clinical severity of SCD [8]. Contrary to this relationship of SCD potentially exacerbating HIV outcomes, some studies have indicated that SCD may protect against HIV infection [11, 12] and others suggested an improvement in HIV progression and outcomes in individuals with SCD [9, 13]. Therefore, additional studies are needed to elucidate whether the interaction between the two disease states might impact both HIV and SCD clinical outcomes. Moreover, potential associations between HIV and SCD and the other acute and chronic manifestations and laboratory features of SCD have not been studied in detail. In this study, we evaluated the association of HIV infection with clinical and laboratory characteristics of patients with SCD from Brazil. In addition, we described HIV outcomes in patients with SCD and HIV.

## Methods

### Study design and setting

This nested case-control study is part of the Recipient Epidemiology and Donor Evaluation Study (REDS-III) Brazilian SCD cohort which is a collaboration between Vitalant Research Institute in San Francisco, CA, USA and six comprehensive sickle cell centers in Brazil: 1) Fundação Hemominas in Belo Horizonte (HBH), Juiz de Fora (JFO), and Montes Claros (MOC), in the Minas Gerais state; 2) Hemorio in Rio de Janeiro, Rio de Janeiro state; 3) Fundação Hemope in Recife, Pernambuco state; and 4) The Child Institute at Hospital das Clínicas da Faculdade de Medicina da Universidade de São Paulo (HCFMUSP) in São Paulo, São Paulo state. Details of the design and recruitment procedures for the REDS-III SCD cohort have been published previously [14]. The REDS-III Brazilian SCD cohort was conducted as part of the international component of the multi-center USA National Institutes of Health, National Heart, Lung, and Blood Institute (NHLBI) REDS-III program that conducts research focused on the safety and adequacy of the blood supply and impact of blood transfusion in recipients in the USA, Brazil, China and South Africa [15].

### Study participants and procedures

The REDS-III infrastructure was utilized to identify eligible patients for this study. As HIV is relatively rare in the SCD population in Brazil [8, 11], HIV positive individuals were actively searched among all SCD patients treated at the REDS-III participating sickle cell centers (not limited to patients enrolled in the REDS-III SCD cohort) in order to include the maximum possible number of HIV positive patients. In order to limit survival bias, all patients with SCD and HIV diagnosed during the 10 years prior to the beginning of this study were considered eligible, whether alive or deceased. Individuals with SCD followed at participating sickle cell centers are screened regularly for HIV, and databases of serology testing were used to identify patients who were HIV positive, alive or deceased. Additionally, all individuals enrolled in the REDS-III SCD cohort were tested for HIV as part of that study.

Living HIV positive patients receiving care at the participating centers were recruited at routine center visits from 2013 to 2015. Details of the study were explained and interested subjects who signed the informed consent were interviewed and had a blood specimen collected. Medical records were abstracted for all living patients who consented and those HIV positive SCD patients who were deceased.

The REDS-III SCD cohort was utilized to identify HIV negative SCD patients to serve as HIV negative controls in the comparison of SCD outcomes between HIV positive and HIV negative SCD patients. HIV-participants in the REDS-III SCD cohort were matched 4:1 to HIV positive patients with SCD on gender, age (± 5 years), center, and SCD genotype. When more than four potential controls were available for any-one HIV positive patient with SCD, the HIV negative participants were randomly selected from all identified potential candidates.

### Eligibility criteria

Inclusion criteria for HIV positive participants were: 1) diagnosis of SCD; 2) diagnosis of HIV (per the medical record and/or per the HIV-test obtained for REDS-III SCD cohort participants); and 3) at least one year of outpatient follow-up by a REDS-III center in the past 10 years. Inclusion criteria for HIV negative participants included 1) inclusion in REDS-III SCD cohort 2) documentation of negative HIV serology and 3) met matching criteria of age, gender, center, and SCD genotype.

### Ethical review

Ethical approval, including a waiver of consent for deceased patients, was granted by the University of California, San Francisco Institutional Review Board (IRB), the Brazilian National Ethical Committee for Research and each participating center IRB. All living participants gave written informed consent for participation.

### Measurements

#### Data collection instruments

A structured interview, and medical record data abstraction were performed for all consenting subjects at enrollment. For deceased participants, only medical record data abstraction was performed. For HIV positive participants, medical record abstraction included both SCD and HIV outcomes data. The interview captured comprehensive demographic data. Additionally, a self-administered audio computer-assisted self-interview (ACASI-QDS Software, Nova Research Co, Bethesda, MD) containing a survey to evaluate sexual and other risk behaviors related to HIV transmission was administrated to HIV positive participants older than 18 years of age [16]. Medical records were reviewed by hematologists or research nurses under the supervision of hematologists, and clinical, laboratory, and treatment data were abstracted using standardized definitions of the clinical manifestations of SCD [17]. The longitudinal retrospective data collection included key SCD or HIV outcomes from the participants’ entire life, from birth to the day that medical record was reviewed. Details of hospitalizations (indication and length of stay), blood transfusions (indications) and laboratory data collected in the year prior to enrollment were also abstracted. Participants not included in the REDS-III SCD cohort, but included in this study to describe HIV positive outcomes in SCD followed the same clinical protocols for SCD management as those included in the cohort, and data collection of those participants followed the same methods briefly described above and previously described in detail [14]. Medical records abstraction of deceased patients was performed using the same instruments used for the other participants. The laboratory data used in the analyses were performed in the year prior to study enrollment, therefore all laboratory values were obtained after HIV diagnosis in HIV positive participants. A comprehensive electronic database was created to centralize all clinical, laboratory, and management information [14].

#### Laboratory methods

Laboratory testing to confirm HIV status of live HIV positive participants and all REDS-III SCD cohort participants was performed at Fundação Pró-Sangue, São Paulo, Brazil. The Architect HIV Ag/Ab Combo (Abbott Diagnostics, Abbott Park, IL, USA) chemiluminescent immunoassay was used for screening. A second test using the Genscreen Ultra HIV Ag-Ab enzyme immunoassay (Bio-Rad, Redmond, WA, USA) was performed in all samples that had a positive screening test. Western blot HIV 1/2 BLOT 2.2 (MP Biomedicals Asia Pacific Pte Ltda, Singapore) was used for confirmation of HIV infection. All tests were performed and interpreted in accordance with the manufacturer’s instructions.

HIV viral load measurement was performed following a previously published procedure [18] on blood samples collected at enrollment of all living participants with HIV positive. Drug resistance testing was conducted by Sanger sequencing.

### Statistical analysis

Our principal outcomes of interest to compare between HIV positive and HIV negative SCD participants were key SCD clinical manifestations - acute chest syndrome, cholecystitis, sepsis/bacteremia, meningitis, splenic sequestration, acute renal failure, chronic renal failure, pyelonephritis, osteomyelitis, avascular necrosis, ischemic stroke, hemorrhagic stroke, abnormal transcranial Doppler (TCD), retinopathy, priapism, leg ulcers, pulmonary hypertension, number of sickle cell pain crises in the year before enrollment in the study, and hospitalizations the year before enrollment. Each clinical outcome was analyzed individually in addition to comparing the occurrence of at least one of these outcomes between HIV positive and HIV negative SCD participants. In addition, composite outcomes were created by grouping outcomes in common clinical categories together. Any infections (acute chest syndrome/pneumonia, pyelonephritis, osteomyelitis, sepsis/bacteremia, or meningitis) were analyzed together as one infectious disease outcome. In the same way, the occurrence of at least one ischemic stroke or an abnormal TCD were analyzed together as a combined neurologic outcome called ischemic stroke/abnormal TCD. The occurrence of at least one of any clinical outcomes known to occur at increased frequency in HIV positive individuals (pneumonia, pyelonephritis, osteomyelitis, sepsis/bacteremia, meningitis, ischemic stroke, avascular necrosis, pulmonary hypertension, chronic renal failure) were analyzed together as a combined outcome called HIV-related complications. Secondary outcomes were SCD laboratory parameters [hemoglobin (Hb), white blood cell (WBC), platelets, reticulocyte, creatinine, lactate dehydrogenase (LDH), total and direct bilirubin, and fetal hemoglobin (HbF)]. The primary predictor variable was HIV diagnosis.

Continuous variables were presented as means with standard deviation (SD), and compared with the two-tailed Student’s t test. Categorical variables were presented as proportions and compared with chi-squared test or Fisher’s exact test. For time to onset of SCD clinical manifestations, we used cox proportional hazards regression analysis with HIV status as the time-varying covariate, stratified by matched group (gender, age, center, SCD genotype) [19], taking into account that clinical manifestations may have occurred prior to the HIV diagnosis. The time of HIV diagnosis was used to adjust time-varying HIV status. Time was defined as the time from birth to first event, or the end of study follow-up (date of medical record review) if they did not have the event of interest, in which case they were right censored. The proportional hazard assumptions were assessed using the Schoenfeld test for non-proportional hazards. If the model would not fit (due to small numbers), we performed a chi-squared test for a crude assessment. Poisson regression models were used for all count outcomes (number of hospital admissions in previous year and number of sickle cell pain episodes in previous year) and a random effect for matching group to determine the incidence risk ratio. All analyses were conducted using Stata 15.1 (Stata Corp., College Station, TX, USA). For the composite outcomes, a *p*-value < 0.05 was considered significant. For clinical manifestations, we used a Bonferroni correction for 21 tests (*p* < 0.0024), and for laboratory data we corrected for 9 tests (*p* < 0.0056).

## Results

### Participants’ descriptions

**Fig. 1:**
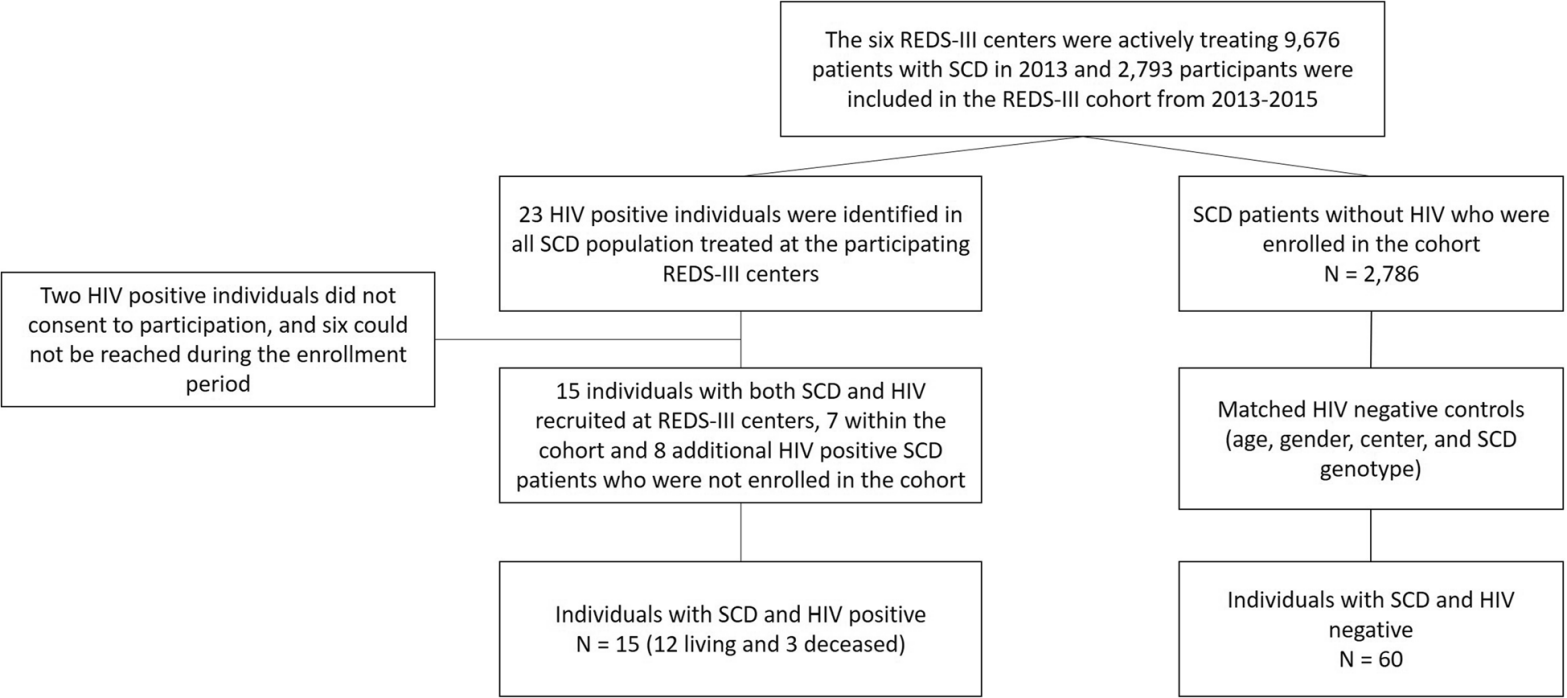
Study sampling. The six Recipient Epidemiology and Donor Evaluation Study (REDS-III) centers were actively treating 9676 sickle cell disease (SCD) patients in 2013 and 2793 were included in the cohort from 2013 to 2015. Out of 23 with SCD and HIV infection identified in the participating centers, 15 were included in this study; of these, 7 were participating in the SCD cohort and 8, were not. Sixty HIV negative participants included in the cohort matched by sex, age, REDS-III center, and SCD genotype were randomly selected to compare SCD outcomes between HIV positive and HIV negative individuals

The six participating centers were actively treating 9676 SCD patients in 2013 and 2793 participants were included in the SCD cohort from 2013 to 2015. The total number of patients treated in the ten years prior to 2013 was not known, but we identified 23 HIV positive SCD patients at the six participating centers in this time period (3 were deceased). Out of 23 HIV positive SCD patients identified, two individuals did not consent to participation in this study, and six could not be reached during the enrollment period. Therefore, we included 15/23 (65.2%) HIV positive participants in this study, 7 within the REDS-III SCD cohort and 8 additional HIV positive SCD patients who were not enrolled in the cohort but treated at the participating centers (Fig. 1); 12 (80%) participants were alive and 3 (20%) deceased. Mean age at the time of data abstraction was 30 ± 13 years for the 15 HIV positive SCD participants. Sixty HIV negative participants were selected from the REDS-III SCD cohort for comparison of SCD outcomes. HIV positive and HIV negative groups were similar with respect to the matching variables of age, gender, SCD genotype, and REDS-III center (Table 1). Most HIV positive patients had HbSS (60%; *n* = 9), 53% (*n* = 8) were female, and 60% (n = 9) were from Hemorio (Table 1). Three (20%) among the 15 individuals with HIV received chronic transfusion therapy (CTT), and one (6.7%) was receiving hydroxyurea (HU) treatment. The indication for CTT was stroke prevention in two (66.6%) individuals, and recurrent vaso-occlusive episode (VOE) in one (33.3%) patient. Probably due to the small number of individuals, there was no significant difference between living and deceased HIV positive participants. However, all three deceased HIV positive participants were HbSS.

**Table 1:** Demographic and treatment characteristics of the HIV positive SCD participants and identified HIV negative SCD controls analyzed to describe HIV outcomes and evaluate the association of HIV infection with SCD outcomes in Brazil

	HIV-positive (***n*** = 15)	HIV-negative (***n*** = 60)	P-value
**Characteristics**	n (%) or mean ± SD		
Age (years)^a^	30.0 ± 13.0	30.9 ± 13.5	> 0.99
Gender			> 0.99
Male	7 (47)	28 (47)	
Female	8 (53)	32 (53)	
SCD genotype			> 0.99
Hb SS	9 (60)	36 (60)	
Hb SC	5 (33.3)	20 (33.3)	
Hb Sβ + −thalassemia	1 (6.7)	4 (6.7)	
Hemocenter			0.94
Hemorio	9 (60)	38 (63.3)	
Hemominas-HBH	4 (26.7)	12 (20)	
Hemominas-MOC	1 (6.7)	6 (10)	
Hemope	1 (6.7)	4 (6.7)	
Hydroxyurea therapy			0.27
Yes	1 (6.7)	14 (23.3)	
No	14 (93.3)	46 (46.7)	
Chronic transfusion therapy			0.63
Yes	3 (20)	7 (11.7)	
No	12 (80)	53 (88.3)	

^a^Age at time of enrollment or age when died for participants who had deceased. *SD* standard deviation; *SCD* sickle cell disease; *HBH* Hemocenter of Belo Horizonte; *MOC* Hemocenter of Montes Claros

### HIV characteristics and outcomes in HIV positive participants with SCD

All HIV positive participants except one (14 of 15) received antiretroviral therapy (ART). Efavirenz, Tenofovir, and Stavudine were the most common drugs that had been prescribed. At the time of the HIV diagnosis, 11 (73.3%) participants were asymptomatic, two (13.3%) had acute clinical symptoms (fever and skin rash), one (6.6%) received the diagnosis of acquired immunodeficiency syndrome (AIDS), and for one (6.6%) participant the information was unknown. The history of opportunistic infections among the 15 participants with HIV positive included recurrent bacterial infections (26.7%; 4/15), candidiasis of bronchi, trachea or lungs (6.7%; 1/15), cytomegalovirus (6.7%; 1/15), and recurrent pneumonia (6.7%; 1/15). Data about the suspected mode of HIV transmission were available in the medical records of 11 (73.3%) out of 15 HIV positive participants: 5 (45.5%) heterosexual sex; 2 (18.2%) transfusion; 2 (18.2%) men who have sex with men (MSM); and 2 (18.2%) vertically transmitted infection. The mean of the most recent CD4 count recorded in the charts was 1053 ± 636.04 mm^3^ (8/15 had data available). The viral load was not detected in seven (58.3%) out of 12 live HIV positive participants. The mean viral load was 2.42 ± 1.36 log copies / mL for five participants with virus detected. The identification of HIV subtype in seven participants showed that four had subtype B and three had subtype F; the prevalence of HIV drug resistance among seven HIV positive participants was 14.3% (1/7). *T69TADN* and *K103N* were the point mutations conferring resistance to the non-nucleoside reverse transcriptase inhibitor. The cause of death of three participants HIV positive was heart failure, respiratory failure, and unknown.

### Risk factors for HIV (ACASI data) in HIV positive participants with SCD

Nine (75%) out of 12 alive HIV positive participants were > 18 years and therefore responded to the ACASI questionnaire about risk factors for HIV infection (see Additional file 1). Most participants who responded to ACASI reported being straight/heterosexual (8/9; 88.9%) and had a mean of 13.6 lifetime sexual partners. Most participants (6/9; 66.7%) disclosed 1–2 sexual partners in previous year. No participants disclosed having sex with partners of same gender, although the mode of transmission was reported as MSM in the medical record for one participant who responded to the ACASI questionnaire. One (1/9; 11.1%) participant reported sex with someone who used injected drugs and one (1/9; 11.1%) reported had sex with a man who has had sex with another man. Four (4/9; 44.4%) reported use of illegal drugs, but none disclosed injecting drug use. Most participants reported no tattoo and piercing. One (1/9; 11.1%) participant had been in jail. Out of five participants currently working, one (1/5; 20%) reported a needle stick injury. All participants received at least one transfusion in their lifetime.

### Comparison of clinical data between HIV positive and HIV negative SCD participants

Cox proportional-hazards models demonstrated that HIV positive participants had significantly higher risk of occurrence of composite HIV-related complications (HR = 7.7; 95%CI 1.5–40.2; *P* = 0.02) and any SCD complication (HR = 4.6; 95%CI 1.1–19.6; *P* = 0.04) when compared to the HIV negative group (Table 2). There was trend towards significant higher risk of any infections in participants with HIV positive compared to those HIV negative (HR = 3.5; 95%CI 0.92–13.4; *P* = 0.07). The frequency of individual SCD complications, ACS/pneumonia, sepsis/bacteremia, pyelonephritis, ischemic stroke, hemorrhagic stroke, abnormal TCD, and pulmonary hypertension, were higher in HIV positive participants when compared to HIV negative, although not statistically significant. Cox proportional-hazards models demonstrated that HIV positive participants had no significantly different risk of occurrence of SCD clinical manifestations when compared to the HIV negative group when we analyzed each clinical manifestation separately, albeit with limited statistical power. Likewise, the incidence risk ratio of sickle cell pain in the year prior to enrollment in the study and hospitalizations in the year before enrollment were similar in the HIV positive and HIV negative groups. No participant had acute renal failure or meningitis.

**Table 2:** Association between HIV infection status and clinical outcomes of sickle cell disease

	HIV-positive (n = 15)	HIV-negative (n = 60)	Effect-size measure (95% CI)^**a**^	P-value
**Clinical manifestations**	n (%)			
**Composite outcomes**				
Any SCD complications				
Yes	13(92.9)	45 (76.3)	HR = 4.6 (1.1–19.6)	0.04
No	1(7.1)	14 (23.7)		
HIV-related complications				
Yes	13 (92.9)	40 (67.8)	HR = 7.7 (1.5–40.2)	0.02
No	1(7.1)	19 (32.2)		
Any infections^b^				
Yes	11(78.6)	32 (57.1)	HR = 3.5 (0.92–13.4)	0.07
No	3 (21.4)	24 (42.9)		
**Single outcomes**				
Acute chest syndrome/pneumonia^b^				
Yes	11 (78.6)	31 (57.4)	HR = 2.12 (0.56–8.2)	0.27
No	3 (21.4)	23 (42.6)		
Cholecystitis^b^				
Yes	0 (0)	2 (3.4)		> 0.99^c^
No	15 (100)	57 (96.6)		
Sepsis/Bacteremia				
Yes	1 (6.7)	1 (1.7)		0.36^c^
No	14 (93.3)	59 (98.3)		
Splenic sequestration				
Yes	1 (6.7)	4 (6.8)		> 0.99^c^
No	14 (93.3)	55 (93.2)		
Chronic renal failure				
Yes	1(6.7)	3 (5)		> 0.99^c^
No	14 (93.3)	57 (95)		
Pyelonephritis				
Yes	1 (6.7)	0 (0)		0.20^c^
No	14 (93.3)	60 (100)		
Osteomyelitis				
Yes	0 (0)	1 (1.7)		> 0.99^c^
No	15 (100)	57 (98.3)		
Avascular necrosis				
Yes	2 (13.3)	11 (18.3)		> 0.99^c^
No	13 (86.7)	49 (81.7)		
Ischemic stroke				
Yes	2 (13.3)	4 (6.7)		0.59^c^
No	13 (86.7)	56 (93.3)		
Hemorrhagic stroke				
Yes	1 (6.7)	0 (0)		0.20^c^
No	14 (93.3)	60 (100)		
Abnormal TCD^b^				
Yes	1(25)	2 (11.1)	HR = 1.14 (0.10–12.7)	0.92
No	3 (75)	16 (88.9)		
Proliferative sickle retinopathy^b^				
Yes	0 (0)	9 (25)		0.3^c^
No	6 (100)	27 (75)		
Priapism (males)^b^				
Yes	0 (0)	2 (7.1)		> 0.99^c^
No	7 (100)	26 (92.9)		
Leg ulcers				
Yes	1 (6.7)	7 (11.7)	HR = 1.27 (0.13–12.4)	0.84
No	14 (93.3)	53 (88.3)		
Pulmonary hypertension^b^				
Yes	1 (20)	2 (6.5)		0.37^c^
No	4 (80)	29 (93.5)		
Ischemic stroke/abnormal TCD				
Yes	3 (20)	5 (8.5)	HR = 2.4 (0.39–14.3)	0.34
No	12 (80)	54 (91.5)		
Hospitalizations in previous year				
Yes	4 (26.7)	18 (30)	IRR = 0.5 (0.2–1.0)	0.05
No	11 (73.3)	42 (70)		
Sickle cell pain in previous year				
Yes	2 (13.3)	14 (23.3)	IRR = 0.3 (0.1–1.4)	0.13
No	13 (86.7)	46 (76.7)		

^a^The result of the Cox proportional hazards regression analysis was presented as a priority stratified by matched group; due to insufficient number of events, the cox proportional-hazards model did not fit, and no effect-size measure was presented. ^b^variables with missing values. ^c^Fisher exact test. *CI* confidence interval; *SCD* sickle cell disease; *TCD* transcranial Doppler; *HR* hazard ratio; *IRR* incidence risk ratio; “Any SCD complication” was defined as the presence of any of the following complications during the patient’s lifetime: acute chest syndrome, cholecystitis, sepsis/bacteremia, meningitis, splenic sequestration, acute renal failure, chronic renal failure, pyelonephritis, osteomyelitis, avascular necrosis, ischemic stroke, hemorrhagic stroke, abnormal transcranial Doppler (TCD), retinopathy, priapism, leg ulcers, vaso-occlusive episode, or pulmonary hypertension; “HIV-related complications” was defined as the presence of any of the following complications during the patient’s lifetime: pneumonia, pyelonephritis, osteomyelitis, sepsis/bacteremia, meningitis, ischemic stroke, avascular necrosis, pulmonary hypertension, or chronic renal failure; “Any infections” was defined as the presence of any of the following complications during the patient’s lifetime: acute chest syndrome/pneumonia, pyelonephritis, osteomyelitis, sepsis/bacteremia, or meningitis

### Association of HIV infection with laboratory data

Platelets, reticulocytes, and lactate dehydrogenase levels were higher, and hemoglobin and fetal hemoglobin levels were lower in HIV positive participants than those with HIV negative, although not statistically significant (Table 3).

**Table 3:** Association between HIV infection status and laboratory data among 75 subjects enrolled in the study from Brazil

	HIV-positive (n = 15)	HIV-negative (n = 60)	P-value
**Laboratory parameters**	mean ± SD		
Hemoglobin (g/dL)	9.0 + 2.3	9.8 + 2.1	0.24
White blood cell (10^9^/L)	10.8 + 4.7	10.8 + 5.5	0.99
Platelets (10^9^/L)	410 + 152.7	344 + 114.5	0.09
Reticulocyte (%)	10.9 + 8.2	8.4 + 4.9	0.17
Log Creatinine (mg/dL)	−0.55 + 0.5	−0.45 + 0.6	0.62
Lactate Dehydrogenase (UI/L)	610.9 + 585.9	444.3 + 235.4	0.17
Total bilirubin (mg/dL)	2.51 + 1.8	2.57 + 1.9	0.93
Direct bilirubin (mg/dL)	0.60 + 0.3	0.72 + 1.3	0.77
Fetal hemoglobin (%)	3.3 + 4.5	8.1 + 7.2	0.05

*SD* standard deviation

## Discussion

This study is one of few to longitudinally investigate the association of HIV infection with the clinical phenotype of SCD. The results indicate that HIV infection likely worsens the clinical presentation of SCD, as we demonstrated an increased risk of any SCD complication as a composite outcome in HIV positive compared to HIV negative SCD participants.

HIV and SCD separately are associated with increased risk of infections and other clinical conditions [6, 20], and we did observe a significant synergistic effect in our analysis; participants with both SCD and HIV had higher risk of HIV-related complications and any SCD complications, and a suggestive but not significantly higher risk of any infections compared to those without HIV. This confirms results of other studies that have suggested HIV increases the risk of clinical complications in SCD [9, 10]. Recently, the prevalence of SCD was shown to be significantly lower in infants with HIV infection from Uganda in a large cross-sectional study, suggesting combined deleterious effects and higher mortality in children with SCD and HIV [21, 22]. Therefore, establishment of HIV infection as a modifier of the severity of SCD may lead to more targeted surveillance and the use of therapeutic interventions for prevention of morbidity and mortality as the interaction of the two diseases may result in therapeutic challenges. Accordingly, special care should be given to patients with co-existing SCD and HIV. Lowe et al. reported the case of a 42-year-old woman with HbSC and HIV who was admitted to the hospital five times in one year after the initiation of ART (stavudine and didanosine). ART was postulated to increase the frequency of sickle cell crises [23]. Furthermore, Odera et al. described an 18-month old African girl with SCD and perinatal HIV infection who developed severe hypersensitivity reaction to first-line ART. ART was interrupted and side effects abated. However, on the day prior to the initiation of an alternative regimen of ART, the patient was admitted to the hospital and died from pneumonia [24]. Other reports also described therapeutic challenges in the treatment of patients with SCD and HIV [25, 26].

Although not significant, in our study we observed differences in the laboratory parameters that could indicate that individuals with HIV have more severe SCD phenotype. Overall high levels of hemolysis markers (e.g. reticulocytes and LDH), lower levels of total Hb and HbF and, higher WBC counts have been associated with increased SCD morbidity and mortality. High levels of reticulocytes have been associated with higher risk of ischemic stroke [27], and hemolytic anemia (low Hb and high LDH) has been associated with increased pulmonary hypertension risk [28]. The lower HbF level in HIV positive participants can be attributed to a lower HU therapy usage than HIV negative participants. The observed differences in therapeutic approaches for HIV positive and HIV negative, although not significant, may be due to the absence of treatment guidelines for patients with both diseases which shows that it is safe to use HU together with the other drugs used to manage HIV infection. In addition, although mostly reversible, side effects of hydroxyurea therapy in individuals with SCD, such as neutropenia and thrombocytopenia, could lead to serious consequences for patients with coexisting HIV [29]. Neutropenia and thrombocytopenia have been associated with HIV disease progression [30, 31], including the development of lethal secondary infections [31]. We observed a higher proportion of HIV positive participants on CTT for secondary stroke prevention, probably reflecting a high risk of cerebrovascular disease in those patients with HIV positive.

While our results and others indicate HIV may worsen SCD, multiple studies have demonstrated a lower prevalence of HIV in individuals with SCD than comparison populations [11, 12], and earlier studies described less severe HIV outcomes in patients with both diseases. Godeau et al. reported none of 8 HIV positive SCD patients had progressed to AIDS, even without ART [16]. Bagasra et al. compared 18 HIV positive SCD patients to 36 HIV positive non-SCD controls matched for age, race and gender. They reported 8 (44%) of 18 individuals with SCD were long-term non-progressors (LTNP) compared to 5 (13.9%) of 36 LTNP in controls with an average follow up of 10 years (*p* = 0.0193). Death due to AIDS occurred in 5 (23%) of 18 patients with HIV and SCD vs. 22 (61%) of 36 of controls [13]. In one previous case report, spontaneous resolution of HIV-associated nephropathy in a patient with SCD was described. The improvement of renal function correlated with the decline in viral load [32]. In our study, most participants with HIV were asymptomatic at diagnosis, demonstrated a relatively high CD4 cell count and low or absent viral load, suggesting that most HIV positive participants were under control for HIV. Only one was diagnosed with AIDS and no death due to AIDS occurred in our sample, although one cause of death was unknown. Further studies are needed to elucidate the interaction between the two disease states that might impact HIV outcomes. Speculated mechanisms for lower HIV prevalence and/or progression in SCD has included an inhibition of HIV replication due to the immunologic changes and pro-inflammatory component of SCD pathophysiology, changes in iron metabolism [11], absence of splenic function [13], as well as the CCR5 delta 32 allele frequency that confers resistance against HIV [33]. Additional investigations are warranted to define the pathophysiological mechanism of protection against HIV infection in individuals with SCD, including in vitro and experimental studies to identify possible differences in the amount and structure of CD4 and chemokine-receptors that may affect viral competence to recognize the infection receptors.

Data collected via the interview showed some well-known risk factors for HIV infection were the suspected mode of transmission in our SCD population such as sex with someone who used injected drugs and at least two partners in the past 12 months [34, 35]. However, transmission was also associated with the treatment of SCD though transfusions. Information about the major modes of transmission in the SCD population may enable effective prevention interventions to reduce HIV transmission, especially in areas where HIV and SCD are endemic.

This study has some limitations. First, the low number of individuals with both SCD and HIV may have resulted in a lack of statistical power to detect associations, especially on single clinical complications analysis or for those with missing observations. Multinational studies, including African countries and other nations where both SCD and HIV are prevalent, should be designed to overcome this power limitation. Although we matched HIV positive and HIV negative participants, our results might be confounded by SCD severity due to the phenotype variability within SCD genotypes. Second, our study may have been limited by the retrospective design and potentially missing data captured in medical records. Lastly, the generalization of the study findings is limited as we were unable to include all the patients identified with HIV positive and SCD in the participating centers. Nevertheless, this is the largest study to date to our knowledge to longitudinally assess the association of HIV with SCD phenotypes. In addition, this is the first study to assess the association of HIV with multiple laboratory and clinical characteristics of SCD.

In summary, our study indicates that HIV infection was associated with an increased risk of development of a composite of key SCD clinical manifestations when compared to HIV negative patients with SCD. Also, our descriptive data suggests a less severe clinical presentation of HIV in patients with SCD. We suggest that efforts should be made to define multidisciplinary protocols and guidelines to provide clear instructions on best management practices for individuals with both diseases.

## Supplementary information

**Additional file 1.** Additional Table 1 – Risk factors for HIV infection among HIV-positive subjects with SCD.

## Data Availability

The datasets used and/or analysed during the current study are available from the corresponding author on reasonable request.

## Abbreviations

SCD: Sickle cell disease
HIV: Human immunodeficiency virus
REDS-III: Recipient Epidemiology and Donor Evaluation Study
HBH: Hemominas in Belo Horizonte
JFO: Juiz de Fora
MOC: Montes Claros
NHLBI: National Institutes of Health, National Heart, Lung, and Blood Institute
IRB: Institutional Review Board
Hb: Hemoglobin
WBC: White blood cell
LDH: Lactate dehydrogenase
HbF: Fetal hemoglobin
SD: Standard deviation
CTT: Chronic transfusion therapy
HU: Hydroxyurea
VOE: Vaso-occlusive episode
ART: Antiretroviral therapy
AIDS: Acquired immunodeficiency syndrome
