# Supplementary material for: Association of HIV infection with clinical and laboratory characteristics of sickle cell disease
Source: BMC Infect Dis. 2020 Aug 27;20:638. doi: 10.1186/s12879-020-05366-z (PMC7457248; doi:10.1186/s12879-020-05366-z)
Supplement: Supplementary file 1 — Additional file 1. Additional Table 1 – Risk factors for HIV infection among HIV-positive subjects with SCD. [file 12879_2020_5366_MOESM1_ESM.docx]

Additional table 1 – Risk factors for HIV infection among HIV-positive subjects with SCD

|  | **Participants with HIV-positive  (n = 9)** | |
| --- | --- | --- |
| **ACASI Questions** | **N or mean±SD** | **% or (range)** |
| Marital status |  |  |
| Single, never married | 4 | 44.4% |
| Living together, not legally married | 1 | 11.1% |
| Married | 3 | 33.3% |
| Separated/divorced | 1 | 11.1% |
| Consider yourself to be |  |  |
| Straight/Heterosexual | 8 | 88.9% |
| Gay/Lesbian/Homosexual | 1 | 11.1% |
| How many people have you had sex with in your entire life? | 13.6±10.5 | (1 - 30) |
| Number of sexual partners in previous year (12 months) |  |  |
| 0 | 1 | 11.1% |
| 1-2 | 6 | 66.7% |
| ≥3 | 2 | 22.2% |
| How many different women have you had sex with since you first began having sex?* For men | 17.7±8.7 | (8 - 25) |
| Man Sex with Man* For men | (N = 5) |  |
| 0 | 4 | 100% |
| missing | 1 |  |
| Female Sex with Female* For women | (n = 4) |  |
| 0 | 4 |  |
| How many times have you had vaginal sex in past 12 months? |  |  |
| 1 to 3 times | 2 | 22.2% |
| 4 to 10 times | 1 | 11.1% |
| More than 10 times | 5 | 55.6% |
| Missing | 1 | 11.1% |
| Vaginal Sex Condom Use |  |  |
| Never | 1 | 11.1% |
| Sometimes | 3 | 33.3% |
| Every time | 4 | 44.4% |
| Missing | 1 | 11.1% |
| Anal sex in past 12 months |  |  |
| Never | 4 | 44.4% |
| 1 to 3 times | 3 | 33.3% |
| More than 10 times | 1 | 11.1% |
| Missing | 1 | 11.1% |
| Anal Sex Condom use | (N = 5) |  |
| Never | 1 | 20% |
| Sometimes | 2 | 40% |
| Every time | 1 | 20% |
| Missing | 1 | 20% |
| Have you ever had sex with anyone who used injected drugs? |  |  |
| No | 7 | 77.8% |
| Yes | 1 | 11.1% |
| Don't Know | 1 | 11.1% |
| Have you ever had sex with anyone who tested positive for HIV? |  |  |
| No | 8 | 88.9% |
| Yes | 0 | 0% |
| Don't Know | 1 | 11.1% |
| Have you ever had sex with a man who has had sex with another man? |  |  |
| No | 7 | 77.8% |
| Yes | 1 | 11.1% |
| Don't Know | 1 | 11.1% |
| Have you ever had sex with anyone who received a blood transfusion? |  |  |
| No | 7 | 77.8% |
| Yes | 1 | 11.1% |
| Don't Know | 1 | 11.1% |
| Have you ever had sex with anyone who has hemophilia? |  |  |
| No | 8 | 88.9% |
| Yes | 0 | 0% |
| Don't Know | 1 | 11.1% |
| How often do you drink beer, wine, liquor, or mixed drinks? |  |  |
| Never | 3 | 33.3% |
| 1-3 times per month or less | 3 | 33.3% |
| 1-2 times per week | 1 | 11.1% |
| 3-6 times per week | 2 | 22.2% |
| Have you ever used any non-injected illegal drugs? |  |  |
| No | 5 | 55.6% |
| Yes* | 4 | 44.4% |
| *Drugs Used: | (n = 4) |  |
| Marijuana | 2 | 50% |
| Cocaine (also called blow or crack) | 2 | 50% |
| Shot up injection drugs |  |  |
| no | 9 | 100% |
| Have you ever injected any non-prescription substances including vitamins, anabolic steroids, or hormones? |  |  |
| No | 6 | 66.7% |
| Yes* | 3 | 33.3% |
| Have you ever shared needles or syringes with another person* | (n = 3) |  |
| no | 3 | 100% |
| Have you ever had a blood transfusion? |  |  |
| No | 0 | 0% |
| Yes | 9 | 100% |
| Transfusion number | 16.7±19.5 | (1 - 50) |
| Have you ever had na organ or tissue transplant? |  |  |
| no | 9 | 100% |
| Have you ever had a surgery? |  |  |
| No | 2 | 22.2% |
| yes | 7 | 77.8% |
| Have you ever had a tooth extraction? |  |  |
| Yes | 9 | 100% |
| Have you ever had a dental root treated or surgery in your mouth? |  |  |
| No | 3 | 33.3% |
| yes | 6 | 66.7% |
| Were you born to an HIV positive mother? |  |  |
| No | 9 | 100% |
| Breastfed by an HIV positive person |  |  |
| No | 9 | 100% |
| Stuck in Jail, Prison,Detention center |  |  |
| No | 8 | 88.9% |
| Yes | 1 | 11.1% |
| Have you had acupuncture treatments? |  |  |
| No | 9 | 100% |
| How many tattoos do you have on your body? |  |  |
| 0 (No tattos) | 6 | 66.7% |
| 1 | 1 | 11.1% |
| 2 | 1 | 11.1% |
| 3 | 1 | 11.1% |
| How many ear or body piercings do you have? |  |  |
| 0 (No piercings) | 7 | 77.8% |
| 1 | 1 | 11.1% |
| 2 | 1 | 11.1% |
| How many people do you personally know who currently have HIV? |  |  |
| 0 (none) | 2 | 22.2% |
| 1 | 4 | 44.4% |
| 2 to 4 | 2 | 22.2% |
| 5 or more | 1 | 11.1% |
| How many people do you personally know with sickle cell and HIV? | (n = 7) |  |
| 0 (none) | 5 | 71.4% |
| 1 | 0 | 0% |
| 2 to 4 | 1 | 14.3% |
| 5 or more | 1 | 14.3% |
| Currently Working |  |  |
| No | 4 | 44.4% |
| Yes | 5 | 55.6% |
| In your profession, do you take care of humans or have exposure to their body fluids? | (n = 5) |  |
| No | 5 | 100% |
| Yes | 0 |  |
| In your professional work have you ever had a needle stick injury | (n = 5) |  |
| No | 4 | 80% |
| Yes | 1 | 20% |
| Contact with secretions from other people | (n = 5) |  |
| No | 5 | 100% |
| Yes | 0 | 0% |
| How become infected with HIV |  |  |
| Sex | 4 | 44.4% |
| Transfusion | 2 | 22.2% |
| Don’t know | 2 | 22.2% |
| missing | 1 | 11.1% |
| When do you think you may have been infected with HIV? (Year) |  |  |
| 1980's | 1 | 11.1% |
| 1990's | 2 | 22.2% |
| 2000's | 1 | 11.1% |
| 2010's | 2 | 22.2% |
| Don’t know | 2 | 22.2% |
| missing | 1 | 11.1% |
| Are you currently taking antiretroviral therapy? |  |  |
| No | 1 | 11.1% |
| Yes | 7 | 77.8% |
| missing | 1 | 11.1% |
